# Most infants with prenatal osteogenesis imperfecta diagnosis and poor prognosis survive: experience of a quaternary care osteogenesis imperfecta center

**DOI:** 10.1093/jbmrpl/ziaf022

**Published:** 2025-02-02

**Authors:** Ricki S Carroll, Sarah Little, Tina McGreal, Shannon Bonner, Daria Willis, Jeanne M Franzone, Jeffery Campbell, Margaret Chou, Megan B Raymond, Andrea Schelhaas, Joanna Costa, Mahim Jain

**Affiliations:** Division of Orthogenetics, Department of Pediatrics, Nemours Children's Hospital, Wilmington, DE 19803, United States; Sidney Kimmel Medical College, Thomas Jefferson University, Philadelphia, PA 19107, United States; Division of Orthogenetics, Department of Pediatrics, Nemours Children's Hospital, Wilmington, DE 19803, United States; Division of Orthogenetics, Department of Pediatrics, Nemours Children's Hospital, Wilmington, DE 19803, United States; Division of Orthogenetics, Department of Pediatrics, Nemours Children's Hospital, Wilmington, DE 19803, United States; Sidney Kimmel Medical College, Thomas Jefferson University, Philadelphia, PA 19107, United States; Sidney Kimmel Medical College, Thomas Jefferson University, Philadelphia, PA 19107, United States; Department of Orthopedics, Nemours Children’s Hospital, Wilmington, DE 19803, United States; Sidney Kimmel Medical College, Thomas Jefferson University, Philadelphia, PA 19107, United States; Division of Neurosurgery, Department of General Surgery, Nemours Children’s Hospital, Wilmington, DE 19803, United States; Sidney Kimmel Medical College, Thomas Jefferson University, Philadelphia, PA 19107, United States; Advanced Delivery Program, Department of General Surgery, Nemours Children’s Hospital, Wilmington, DE 19803, United States; Sidney Kimmel Medical College, Thomas Jefferson University, Philadelphia, PA 19107, United States; Department of Obstetrics and Gynecology, Thomas Jefferson University Hospital, Philadelphia, PA 19107, United States; Advanced Delivery Program, Department of General Surgery, Nemours Children’s Hospital, Wilmington, DE 19803, United States; Sidney Kimmel Medical College, Thomas Jefferson University, Philadelphia, PA 19107, United States; Advanced Delivery Program, Department of General Surgery, Nemours Children’s Hospital, Wilmington, DE 19803, United States; Division of Neonatology, Department of Pediatrics, Nemours Children’s Hospital, DE, United States; Division of Orthogenetics, Department of Pediatrics, Nemours Children's Hospital, Wilmington, DE 19803, United States; Sidney Kimmel Medical College, Thomas Jefferson University, Philadelphia, PA 19107, United States

**Keywords:** osteogenesis imperfecta, skeletal dysplasia, lethal, prenatal, lung hypoplasia

## Abstract

Osteogenesis imperfecta is a genetic condition with improperly or inadequately produced Type I collagen. Manifestations include bowing deformities, fractures, hydrocephalus, respiratory insufficiency, and feeding difficulty. Moderate or severe OI is often diagnosed prenatally based on ultrasound findings and genetic testing may be labeled as lethal. Here, we present 18 infants with moderate to severely presenting OI who received neonatal care at a single center over a 5 yr period, 10 of which were delivered at our institution. All 18 infants survived to neonatal discharge, with 7 infants requiring respiratory support and 9 infants requiring feeding support at discharge. Through Fisher Exact Test, Mann–Whitney U Test and backward elimination regression, we do not observe that lethal or possibly lethal diagnoses prenatally were correlated with medically relevant outcomes such as need for respiratory support at discharge or need for feeding support at discharge. Sixteen of the eighteen individuals are alive, with a minority requiring either respiratory or feeding support. With a multidisciplinary team approach to neonatal care, outcomes may be optimized. Infants formerly diagnosed with lethal OI may survive. Given our findings, and lack of correlation of prenatal assessments with survival and other medical outcomes, we recommend all families be given the option to pursue medical interventions.

## Introduction

OI is a heterogenous genetic bone condition that occurs in approximately 1 in 15 000 to 20 000 live births.^1–3^ Clinical manifestations of OI are typically due to either inadequately or improperly produced type I collagen and result in a broad range of clinical severity, even among family members.^3^ Notable orthopedic manifestations include bone deformity and fragility with a predisposition to fractures following minimal or absent trauma. Short stature may also be observed, often occurring in moderately to severely affected individuals. Other nonskeletal features may include blue or gray sclerae, joint hypermobility, dentinogenesis imperfecta, hearing loss, a propensity for easy bruising, respiratory and feeding difficulties, and hydrocephalus.^4^^,^^5^ Individuals with OI have typical intellect.^6^ Though there is variation in physical health-related quality of life data for individuals with OI, mental health and psychosocial quality of life are reported to be the same or better when compared with unaffected individuals.^7^^,^^8^

Individuals with OI are often classified into 1 of 4 subtypes: type I (mild/nondeforming), type II (perinatal lethal), type III (severe/progressively deforming), and type IV (moderately deforming); however, this classification system continues to evolve with increasing knowledge.^5^^,^^9^ Those with a mild phenotype are often diagnosed postnatally or in the pediatric setting after experiencing multiple unexplained fractures. Concerns for moderate to severely presenting OI are often noted in utero when fractures, shortening, and/or bowing of the long bones are found on prenatal ultrasound.^3^

When OI is suspected and/or molecularly confirmed in the prenatal period, families may be counseled that the diagnosis is lethal or severely life-limiting based on prenatal ultrasound observations and previously reported genotype-phenotype correlations.^10^^,^^11^ Ultrasound parameters for predicting lethality in skeletal dysplasias have been studied and include the chest-to-abdominal circumference ratio of <0.6 and femur length-to-abdominal circumference ratio (FL/AC) of <0.16.^11–13^ However, there are nuances to this strategy, for instance in cases where bowing deformities and fractures limit the accuracy of true femur length measurements.^14^ While genotype-phenotype correlations are also considered when predicting lethality, there can be a range of clinical variability even among those with the same genotype.^3^^,^^4^ Some specialized delivery centers have reported on the accuracy of these methods in predicting lethality, yet many of the pregnancies evaluated are ultimately terminated further limiting the ability to draw conclusions.^15^ These limitations pose a challenge for perinatal providers counseling families on the diagnosis and attempting to prognosticate postnatal survival probability. Consequently, this information can cloud conversations surrounding delivery planning and influence access to potential life-saving therapies including invasive mechanical ventilation and feeding support.

Advancements in medical technology and the option for life-sustaining interventions have significantly altered the prognoses for severely affected infants. Here we describe perinatal outcomes of infants referred to a single specialized center after receiving a prior diagnosis of possibly lethal, lethal, or type II OI where parents sought medical intervention after birth. We also outline advances in respiratory and feeding support needs, as well as length-of-stay for these neonates. The success of this multidisciplinary approach to neonatal OI care both challenges previously defined expectations for this patient population and offers a chance at survival.

## Materials and methods

### Study design

Using patient lists created within our electronic medical record, we identified individuals with OI who have received care in our quaternary care neonatal intensive care unit (NICU), from September 2019 to August 2024. Inclusion criteria were any infant with OI who received care in the NICU, regardless of birth hospital. Excluded were patients without a diagnosis of OI and those who did not receive care in the NICU. After refining the list to 18 individuals, we completed a comprehensive chart review including prenatal, birth, neonatal, pediatric medical, and surgical histories. We specifically examined ratios at second trimester anatomy ultrasound including FL/AC and thoracic circumference to abdominal circumference (TC/AC) when available, as well as qualitative narrative comments. Genetic variants were reviewed in ClinVar for prior description of lethality association.^16^ This work is being completed under IRB approval 2123944 through Nemours Office of Human Subjects Protection.

### Statistical methods

Differences in the demographic characteristics were assessed using Fisher Exact Test. The Mann–Whitney U test was used to determine the differences between median birth weight, birth weight percentile, and length of hospital stay. Birth weight percentile was calculated based on reported gestational age.^17^ The proportions of individuals with respiratory support at discharge, feeding support at discharge, and VP shunt placement were analyzed using the Fisher Exact Test. In order to further examine the correlation between lethal/possibly lethal diagnosis of OI and outcomes (respiratory support at discharge, feeding support at discharge, VP shunt, and length of hospital stay), we utilized a logistic regression by applying a backward-elimination strategy where all variables were included in a full model and at each iteration an independent variable that did not improve the model was removed. Backward elimination was stopped when all included variables led to model improvement. In the model, sex, respiratory support at discharge, feeding support at discharge, and VP shunt were included as binary variables, and birth weight percentile and length of stay as continuous. In order to identify potential correlations, we examined prenatal diagnostic classification and need for support at discharge as dependent variables.

## Results

### Prenatal lethal or possibly lethal diagnosis did not correlate with survival

Our clinical experience involves 18 infants over the last 5 yr (Table 1). Of these, 12 were provided a lethal or possibly lethal diagnosis in utero, 4 were provided a nonlethal diagnosis, and 2 are unknown. For 4 infants, a prenatal lethal diagnosis was suspected based on genetic findings of glycine substitution variants in *COL1A1* or *COL1A2* that were previously published as causing lethal OI.^18^^,^^19^

**Table 1:** Genetic description of cohort with prenatal lethality assessment.

**Patient number**	**Gene**	**Variant**	**Previously reported as lethal**	**TC/AC**	**FL/AC**	**Prenatally counseled as lethal/possibly lethal**
**1**	*COL1A2*	c.1711G>A (p.G571R)	No	Not available	Not available	Yes
**2**	*COL1A1*	c.2533G>A (p.G845R)	Yes	0.85	0.13	Yes
**3**	*COL1A2*	c.839G>A (p.G280D)	No	Not available	Not available	Yes
**4**	*LEPRE1*	c.1080+1G>T (homozygous)	No	Not available	Not available	No
**5**	*COL1A1*	c.1273G>A (p.G425S)	Yes	Not available	Not available	Yes
**6**	*COL1A1*	c.4327G>A (p.A1443T)	No	Not available	Not available	Not applicable^a^
**7**	*COL1A2*	c.1199G>A (p.G400N)	No	0.73	0.12	Yes
**8**	*COL1A1*	c.1156G > A (p.G386R)	No	0.85	0.16	Yes
**9**	*COL1A1*	c.2155G>A (p.G719S)	No	Not reported	0.12	Yes
**10**	*COL1A1*	c.1462-3C>A	No	Not reported	0.17	Yes
**11**	*COL1A1*	c.3118G>A (p.G1040S)	No	Not reported	0.18	No
**12**	*COL1A1*	c.994G>A (p.G332R)	No	0.91	0.15	No
**13**	*COL1A2*	c.3170G>A (p.G1057D)	No	Not available	Not available	Not applicable^a^
**14**	*COL1A2*	c.1072G>A (p.G358S)	No	Not available	Not available	No
**15**	*COL1A2*	c.2845G>A (p.G955N)	No	0.78	Not reported	Yes
**16**	*COL1A1*	c.4237G>A (p.D1413N)	Yes	Not reported	0.16	Yes
**17**	*COL1A2*	c.3269G>A (p.G1090D)	Yes	0.93	0.17	Yes
**18**	*COL1A2*	c.1883G>A (p.G638D)	No	0.85	Not reported	Yes

a Prenatal counseling for OI not applicable to patient 6 or 13 as OI diagnosis was unknown prenatally.

Abbreviations: FL/AC, femur length to abdominal circumference; TC/AC, thoracic circumference to abdominal circumference.

In 4 cases there were ultrasound findings of FL/AC < 0.16. There were no identified cases with TC/AC < 0.6; however, thoracic circumference was not reported for 4 cases where anatomy ultrasound was available. In cases with an unknown lethality assessment prenatally, the first (patient 6, Tables 1 and 2) was not suspected to have OI prenatally and came to clinical attention with fractures in bilateral femurs and right scapula at 1 mo of age. He was subsequently diagnosed with OI and treated in the NICU. In the second case, (patient 13, Tables 1 and 2), there was a prenatal diagnosis of OI but there was no information on lethality assessment from available prenatal records. All individuals survived neonatal admission to hospital discharge. Two individuals died after discharge at age 7 mo (patient 13) and at age 13 mo (patient 5). Patient 13 presented with worsening respiratory failure in the setting of acute viral illness and required intubation, with subsequent complications of a pneumothorax. During this admission she suffered a cardiac arrest requiring chest compressions with successful resuscitation, and the family ultimately decided to withdraw technology-based care. For patient 5, family goals of care changed after decompensation from acute respiratory illness. The child went home on hospice from a local facility and later died due to acute respiratory arrest.

**Table 2:** Neonatal course for cohort.

**Patient number**	**Gestational age (wk)**	**Birth weight (grams)**	**Birth weight percentile**	**Age at first bisphosphonate infusion (d)**	**Age at discharge (d)**	**Respiratory support at discharge**	**Feeding support at discharge**	**VP shunt**
**1**	37 1/7	2500	8.1	7	70	CPAP	NG Tube	No
**2**	38 5/7	2750	7.5	7	46	CPAP	NG Tube	No
**3**	33 4/7	1820	5.9	27	112	CPAP	G Tube	No
**4**	35 5/7	2993	83	110	237	None	NG Tube	Yes
**5**	37	2010	<1	15	189	Nasal cannula	G Tube	No
**6**	39	3045	19.1	72	102	None	None	No
**7**	38 4/7	2685	6.4	10	80	None	None	No
**8**	37 4/7	2180	<1	10	366	Tracheostomy	G Tube	Yes
**9**	38 3/7	2870	14.7	6	14	None	None	No
**10**	37 5/7	2560	7	15	19	None	None	No
**11**	37 5/7	1910	<1	6	46	None	None	No
**12**	37 1/7	2500	8.1	14	57	Nasal cannula	NG Tube	No
**13**	37	2000	<1	19	104	CPAP	G Tube	Yes
**14**	38	3430	74.8	8	18	None	None	No
**15**	36 3/7	1565	<1	16	58	None	None	No
**16**	38 2/7	2710	8.3	9	39	None	None	No
**17**	35 6/7	2520	27.1	14	42	None	None	No
**18**	39 4/7	2980	9.4	33	73	None	NG Tube	No

In examining correlations between prenatal diagnosis with gestational age, birth weight percentile, length of hospital stay, respiratory support at discharge, feeding support at discharge, or need for VP shunt, we did not identify significant differences between groups (Tables 2 and 3). Overall, 4 individuals were born prior to 37 wk (2 preterm labor, 1 preeclampsia, 1 elective palliative), with 1 in the nonlethal prenatal diagnosis group and 3 in the lethal/possibly lethal group. We observed 5 individuals who were <1%ile for birth weight based on gestational age, with 1 in the nonlethal group, 3 in the lethal/possibly lethal group, and 1 in the unknown group. All infants received bisphosphonates at a median age of 14 d (range 6-110 d). Seventeen of the eighteen received pamidronate and 1/18 received zoledronate (patient 18, Tables 1 and 2). The infant who received zoledronate did so at another facility and had complications of hypocalcemic seizure, which was not observed in the group that received pamidronate. In the backward elimination logistic regression analysis, we do not observe significantly correlated variables, including birth weight percentile, length of hospital stay, or need for support at discharge, to prenatal lethal/possibly lethal group.

**Table 3:** Comparison of lethal/possibly lethal group versus the moderate/severe group.

	**Lethal/possibly lethal**	**Moderate/severe**	***p* value**
**Number**	12	4	
**Female sex**	4	2	.60
**Gestational age (yr), median (range)**	37 5/7 (33 4/7-39 4/7)	37 5/7 (35 6/7-38)	.67
**Birth weight g, median (range)**	2560 (1565-2980)	2465 (1910-2520)	.33
**Birth weight percentile median (range)**	7 (<1-14.7)	17.6 (<1-74.8)	.43
**Length of stay, days median (range)**	70 (14-366)	44 (18-57)	.15
**Respiratory support at discharge**	5	1	1
**Feeding support at discharge**	7	1	.57
**VP shunt**	2	0	1

### Length of hospital stay correlates with VP shunt need

We did not observe statistically significant differences in the length of hospital stay, with the lethal/possibly lethal group having a median of 70 d (range 14 to 366 d) and the nonlethal group having 44 d (range 18-57 d), *p* = .15 (Tables 2 and 3). In examining infants that required hospital stays greater than 60 d, none in the nonlethal group required that length of stay, while 7 in the lethal/possibly lethal group required a length of stay greater than 60 d (*p* = .09). For 2 individuals in the lethal/possibly lethal group, there was a hospital stay of greater than 200 d, and both required VP shunt, respiratory support at discharge, and feeding support at discharge. In the regression analysis, we observe a significant correlation for length of hospital stay with presence of VP shunt (*p* < .001), with additional trend for significance with low-birth-weight percentile (*p* = .061) and respiratory support at discharge (*p* = .053).

### Respiratory support correlates with feeding support at discharge

In total 7 individuals required respiratory support at discharge (5 in the lethal/possibly lethal group, 1 in the nonlethal group, 1 in the unknown group), and 9 individuals required feeding support at discharge (7 in the lethal/possibly lethal group, 1 in the nonlethal group and 1 in the unknown group, Tables 2 and 3). In the regression analysis, we observe a significant correlation between need for respiratory support at discharge and feeding support at discharge (*p* < .001). In addition, the need for respiratory support at discharge correlates with low-birth-weight percentile (*p* = .05).

### Most individuals with poor prenatal prognosis are living without respiratory or feeding support and are mobile

The 16 living individuals have a median current age of 23.5 mo (range 2-58 mo, Table 4). Of the 7 individuals that required respiratory support at discharge, 5 were diagnosed with lethal/possibly lethal OI. Of the 12 infants diagnosed with lethal/possibly lethal OI prenatally, 1 was discharged on nasal cannula, 3 were discharged on nasal CPAP, and 1 with a tracheostomy and ventilator; the other 7 were discharged on room air. Currently 2 (ages 29 and 50 mo) have tracheostomies, and the remainder do not require respiratory support. One of the individuals with a tracheostomy had been discharged on CPAP, but due to increasing respiratory support, tracheostomy was pursued at 6 mo of age. For those who were weaned off respiratory support, it was discontinued between 9 and 14 mo of age (Table 5).

**Table 4:** Summary of current status of the cohort.

	**Entire cohort**	**Lethal/possibly lethal**	**Genetic variant previously reported as lethal**	**FL/AC < 0.16**
**Living**	16/18	11/12	3/4	4/4
**Current age median, mo (range)**	23.5 (2-58)	23 (2-58)	6 (5-47)	23.5 (11-47)
**Respiratory support**	2/16	2/11	0/3	0/4
**Feeding support**	5/16	4/11	1/3	1/4
**Bearing weight on lower extremities (>2 yr old)**	5/8	3/5	1/1	2/2

Abbreviation: FL/AC, femur length to abdominal circumference.

**Table 5:** Duration of respiratory and feeding support for individuals with lethal/possibly lethal OI.

**Patient number**	**Respiratory support at discharge**	**Current respiratory support**	**Age at discontinuation of respiratory support**	**Feeding support at discharge**	**Current feeding support**	**Age at discontinuation of feeding support**
**1**	CPAP 5 0.30	None	14 mo	NG-tube	None	6 mo
**2**	CPAP 8 0.21	None	12 mo	NG-tube	G-tube	Remains supported at 3 yr old
**3**	CPAP 12 0.30-0.40	Tracheostomy	Remains trach/vent dependent at 4 yr old	G-tube	GJ-tube	Remains supported at 4 yr old
**5**	1 L NC	Not applicable^a^	Remained supported until death	G-tube	Not applicable^a^	9 mo
**7**	None	None	Not applicable	None	None	Not applicable
**8**	Tracheostomy	Tracheostomy	Off vent during day at 2 yr 5 mo of age	G-tube	G-tube	Remains supported at 2 yr old
**9**	None	None	Not applicable	None	None	Not applicable
**10**	None	None	Not applicable	None	None	Not applicable
**15**	None	None	Not applicable	None	None	Not applicable
**16**	None	None	Not applicable	None	None	Not applicable
**17**	None	None	Not applicable	None	None	Not applicable
**18**	None	None	Not applicable	NG-tube	NG-tube	Remains supported at 10 wk of age

a Current respiratory and feeding support not applicable to patient 5 as he is deceased.

Of the 9 individuals requiring feeding support at discharge, 6 were diagnosed with lethal/possibly lethal OI. Of the 12 infants diagnosed with lethal/possibly lethal OI prenatally, 3 were discharged with an NG-tube, 3 were discharged with a G-tube, and 6 were discharged eating by mouth. Currently, 5 individuals continue to require enteral support, 4 via G-tube and 1 via NG-tube. For those who were weaned off feeding support, it was discontinued between 2 and 9 mo of age (Table 5).

Five children have undergone lower extremity realignment/rodding (median 36 mo; range 14-39 mo), and 1 has undergone upper extremity realignment/rodding (53 mo). Of the 8 individuals older than 2 yr old, 1 is walking without support (age 44 mo), 1 is walking with support (age 39 mo), 1 scoots, stands with support, and is independent in a power wheel chair (age 58 mo), 2 are crawling or scooting and standing with support (ages 24 and 47 mo), and the remaining 3 are rolling (25, 29, and 50 mo).

**Figure 1:**
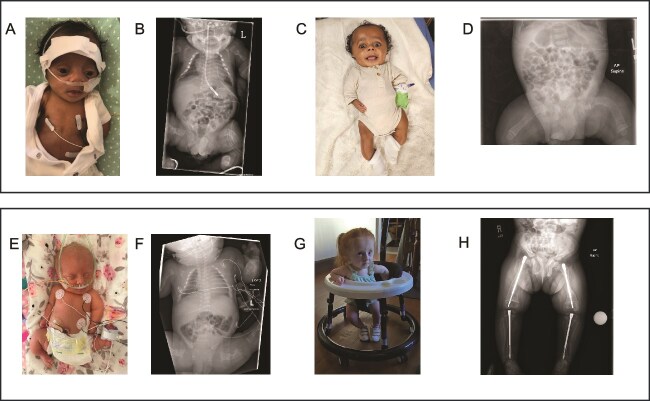
Clinical and radiographic progression of 2 infants with variants previously associated with lethal OI. (A) Patient 15 at 1 mo of age; clinical photograph, infant on nasal cannula. (B) Radiograph of patient 15 at 1 wk of age demonstrating demineralized bones, chest wall deformity, multiple healing rib fractures, broad capacious canals of the upper and lower extremities with thin crumpled cortices, multiple healing fractures and bowing deformities. (C) Patient 15 at 8 mo of age; clinical photograph during bisphosphonate infusion, infant awake, alert, spontaneously moving upper and lower extremities. Note limb bowing and blue sclera that are indicative of severe OI presentation. (D) Radiograph of patient 15 at 7 mo of age demonstrating persistent but improved demineralization, bilateral femoral bowing, thickening of cortices, pamidronate lines in the metaphyses. (E) Patient 2 at 1 d old, clinical photograph of infant on nasal CPAP, with feeding support via NG tube and umbilical venous catheter. Bowing and deformity in the lower extremities. (F) Radiograph of patient 2 at day of life 0 demonstrating umbilical venous catheter, thin gracile ribs, crumpled extremity bones with capacious canals, thin cortices and multiple healing fractures. (G) Patient 2 at 4 yr old; clinical photograph demonstrating ability to bear weight on lower extremities. (H) Radiograph of the lower extremities of patient 2 at 3 yr old demonstrating bilateral femoral and tibial telescopic intramedullary rods placed during realignment and intramedullary rodding procedures. With the rods in place, patient 2 is progressing in a stander and bearing weight through the bilateral lower extremities.

Of the 4 individuals with a genetic finding associated with lethal OI, 3 are living at median age 6 mo (range 5-47 mo). None require respiratory support. Patient 15 (Figure 1) was initially referred to hospice care; however, after surviving the first month of life in the NICU without medical technology, family reevaluated goals of care and subsequently pursued full intervention. Patient 2 (Figure 1) is able to bear weight on lower extremities and has feeding support (G-tube). Of the 4 individuals with FL/AC < 0.16, all are living, with median age 23.5 mo (range 11-47 mo). Both individuals who are above age 2 from this group are able to stand, bearing weight on lower extremities.

## Discussion

Advances in prenatal diagnostics offer increasingly more sophisticated information to guide prenatal counseling and perinatal management. Current practice varies widely but commonly relies upon a combination of fetal ultrasound findings and genetic testing to guide prognostication. However, this general methodology for prenatal assessment and prognosis does not appear to accurately predict postnatal outcomes, especially in conditions such as OI, that manifest along a spectrum and lack validated and standardized predictors of severity.

Fetal ultrasound measurements, such as TC/AC or FL/AC, have been proposed as predictive factors for postnatal outcome when a skeletal dysplasia is suspected or confirmed.^11–13^ Historically, lethality has been understood to occur in most skeletal dysplasias as a result of a small chest circumference and resultant pulmonary hypoplasia.^10^ With the advancement in ultrasound use prenatally, lethality criteria were determined by analyzing ultrasound parameters in the setting of infants at risk for pulmonary hypoplasia. These pivotal studies defined a TC/AC < 0.6 and an FL/AC of <0.16 as strongly suggestive of lethality. However, there are significant limitations in applying these ratios to OI. Specifically, it can be challenging to measure long bones accurately in OI due to bowing, deformities, and fractures.^13^ Due to this, infants with OI are either not included in these studies or are noted to be an exception to the accuracy of these predictive ratios.^11–13^ Additionally, in many of these studies where there is evaluation of the predictive value of these measurements for prognostication, the pregnancy was either terminated or a focus was placed on palliative care postnatally, making survivability impossible to assess.

Fetal MRI is suggested to be useful in assessing pulmonary hypoplasia and lethality in skeletal dysplasias.^20–22^ Despite this, none of our 18 patients had an MRI obtained prenatally and this is a limitation to our study; we are not able to comment on fetal MRI findings related to outcome in our cohort. The lack of fetal MRI usage in our cohort may be due to lack of awareness by providers, lack of access, and/or insurance barriers. Recent literature has suggested changes to US and MRI measurement cutoffs to indicate lethality; however, this evolution of standards is not yet widespread in general maternal fetal medicine practice, as evidenced by the counseling our patients received prior to presenting to us. More research is needed to fully understand the utility and accuracy of these image modalities in this specific patient population.

Genetic testing is commonly relied upon in the prenatal setting to assist with prognostication, anticipatory guidance, and medical decision-making for families. When an individual’s genetic variant correlates with a case reported in the literature in which the infant did not survive, the family may be counseled that their infant is likely to have the same outcome. This can influence decision-making toward termination or comfort-focused care. However, the previously described variability in presentation^3^^,^^4^ and our experience presented here, in which 3 out of 4 infants with a variant previously associated with lethal OI survived without need for long-term respiratory support, suggests that relying solely on genetic findings in predicting lethality has limitations.

The ability to accurately predict severity based on ultrasound and genetic criteria is limited because they do not consistently account for the impact of available medical interventions after birth, specifically treatment of respiratory failure related to fracture burden, respiratory splinting due to pain, and chest wall compliance. The predictions can then become a self-fulfilling prophecy if no treatment is administered.^23^ In our cohort, all but 2 expectant parents received a prenatal diagnosis of OI. Of those with a prenatal diagnosis, prognostic counseling prior to referral was based on commonly used fetal ultrasound findings and/or molecular test results, with 4 having molecular testing suggestive of lethality and 4 prognosticated based on ultrasound ratios. In total, 12 of the 18 patients were given the diagnosis of lethal or possibly lethal OI. The families in this cohort pursued care at a specialized neonatal OI center. No infants required chest compressions in the delivery room, and only 1 received intubation and invasive mechanical ventilation in the delivery room. All patients survived to initial hospital discharge, and 16 of the 18 are alive today. The majority of these infants do not rely on breathing or feeding support. Furthermore, while there is a trend, infants with predicted lethal or possibly lethal OI did not demonstrate significantly longer lengths of stay or need for respiratory or feeding support at discharge compared with those predicted to have milder phenotype.

The current nosology of genetic skeletal disorders continues to reference the Sillence classification system, despite advancements in molecular testing, which has expanded our knowledge of current phenotypes.^24^ While our knowledge of OI classification continues to evolve, the application of this current system to predict postnatal survivability remains limited and should be considered with caution. Instead, OI should be considered a condition that presents with a spectrum of postnatal manifestations that can be challenging to predict prenatally. Indeed, at our center, our approach to counseling acknowledges the challenges and uncertainty of prognostication prenatally. The term lethal is avoided in our counseling, and instead, the focus is on the phenotypic spectrum of mild, moderate, or severe. Fetal ultrasound findings and genetic testing can reassure us toward the milder end of the spectrum or be suggestive for a more severe manifestation. Counseling is nondirective, and all options are discussed with plans developed in accordance with a family’s wishes and goals of care.

Our approach to care, ideally, begins with a prenatal assessment, including a multidisciplinary evaluation and counseling within the framework of mild, moderate, and severe OI. Plans for mode of delivery and neonatal care are developed with shared decision-making and based on individualized goals of care. When determining delivery timing, care is taken to interpret fetal growth restriction, umbilical artery dopplers, and antenatal surveillance in the setting of known skeletal dysplasia. In all deliveries, we minimize pincer or single point fulcrum movements and avoid excessive extension or flexion of the neck. In cesarean delivery, a generous low transverse uterine incision is made and amniotomy is delayed to effect en caul delivery. Neonatal management focuses on targeted respiratory support and immediate focused pain control in expected fractures after birth.^25^ Uniquely experienced nursing care ensures minimal handling through clustering of nursing care and assessment and minimizing interventions. We advocate for early use of bisphosphonate therapy, as soon as is practical. Families are supported in early parental engagement and education and ongoing psychosocial support. Early identification of a local medical home for collaboration in long-term care is essential as patients transition from the NICU to long-term multidisciplinary care.

One limitation of this analysis is that diagnosis and prognosis were determined prior to referral. The cohort demonstrated variation in methodology of prognostication, and therefore counseling. Standardization of assessment is needed in future studies to validate the predictive value of any prenatal findings alone or in various combinations. Nevertheless, these observations demonstrate that current prenatal prognostication is not predictive of lethality, especially when considering interventions after birth. Though the numbers are small, we are capturing 18 cases of a rare diagnosis.

Further studies are needed to evaluate the efficacy of prenatal assessments in predicting lethality in OI. We recommend that ultrasound measurements and genetic testing results are utilized with caution in this patient population. In the setting of a prenatal diagnosis of OI, the option for evaluation and counseling at a specialized center with experience in caring for patients with OI through infancy, childhood, and into adulthood should be offered. Given our observations that prenatal assessments are not correlated to medical outcomes, including survival, we recommend all families be given the option to pursue medical interventions.

## Data Availability

The data underlying this article will be shared on reasonable request to the corresponding author.
